# Kinetics and Mechanism of Ternesite Formation from Dicalcium Silicate and Calcium Sulfate Dihydrate

**DOI:** 10.3390/ma15072626

**Published:** 2022-04-02

**Authors:** Xiaofei Huang, Fei Shi, Guoling Wang, Jiangbo Yu, Suhua Ma, Weifeng Li

**Affiliations:** College of Materials Science and Engineering, Nanjing Tech University, Nanjing 211816, China; 201961203147@njtech.edu.cn (X.H.); 202061203251@njtech.edu.cn (F.S.); 201961103078@njtech.edu.cn (G.W.); 202161203298@njtech.edu.cn (J.Y.)

**Keywords:** ternesite, formation mechanism, formation kinetics, activation energy

## Abstract

The kinetics and mechanism of ternesite formation (calcium sulfosilicate, Ca_5_(SiO_4_)_2_SO_4_, C_5_S_2_$) were investigated by studying the reaction between beta-dicalcium silicate (β-C_2_S) and calcium sulfate dihydrate (CaSO_4_∙2H_2_O). Mineralogical composition development was monitored using X-ray diffraction (XRD) and backscattered scanning electron microscopy (BSEM) coupled to energy-dispersive X-ray spectroscopy (EDS). Ternesite can form in the 1100 to 1200 °C range by the solid-phase reaction of β-C_2_S and CaSO_4_. The formation of ternesite is favored by increasing the sintering temperature or extending the sintering time. The solid-phase reaction is carried out by diffusion of CaSO_4_ to β-C_2_S. The kinetics equation of ternesite is consistent with three-dimensional diffusion models (3-D model, D3 model or Jander model). The equation of the D3 model is 1 − 2α/3 − (1 − α)^2/3^ = kt. On the basis of the Arrhenius equation, the activation energy of ternesite is 239.8 kJ/mol.

## 1. Introduction

The Portland cement industry contributes 5% of global CO_2_ emissions due to the breakdown of limestone and the burning of chemical fuels [1]. Sulfoaluminate (CSA) cement is considered an ideal alternative to Portland cement, owing to its low level of energy consumption and low CO_2_ emissions in the manufacturing process [2,3,4,5]. However, the limited supply of aluminum-containing raw materials and their high price restrict the application of CSA cement. Ternesite is a typical intermediate phase in conventional calcium sulfoaluminate (CSA)-type clinker production processes. This mineral is usually formed as an intermediate transition compound in the pre-tropical zone during the preparation of silicate clinker [6,7,8,9,10,11]. In sulfate aluminate cement clinker, it is mainly formed when there is an excessive amount of gypsum dispensed in the raw meal or when the calcination temperature is too low [12,13,14]. When the aluminum phase is present, ternesite (C_5_S_2_$) is a clinker phase with hydration activity. It can bridge the intermediate gap between the fast reaction of ye’elimite and the late strength contribution of belite [15]. Belite–calcium sulfoaluminate–calcium sulfosilicate (BYT) system cement combines the advantages of high early strength of CSA cement and the excellent durability of belite cement. On the other hand, BYT clinker has the following benefits: low sintering temperature, low CO_2_ emissions, and easy availability of raw materials [16]. Therefore, BYT system cement have become the focus of global research in recent decades.

The formation of ternesite has been studied by several authors [17,18,19]. Ternesite can be formed as an intermediate phase in CSA cement clinker at 900 °C; it decomposes above 1200–1280 °C to C_2_S and CaSO_4_ [20]. Jing G. [21] systematically analyzed the kinetics of C_5_S_2_$ formation by different C_2_S polycrystals, and the results showed that β-C_2_S is more likely to form C_5_S_2_$. K. Ben Addi et al. [22] studied the mineralogical evolution of sulfoaluminate clinker and found that ternesite formed at 800 °C, and the clinker, which contained ye’elimite, belite, and ternesite, formed at temperatures between 1100 and 1250 °C, was stable. W. Gutt et al. [23] prepared pure calcium silicosulfate by sintering mixtures of calcium carbonate, crushed quartz, and CaSO_4_∙2H_2_O for 150 h at 1150 °C. Y. B. Pliego-Cuervo [24] synthesized calcium silicosulfate by heating a mixture of beta-dicalcium silicate (β-C_2_S) and CaSO_4_ at 1100 °C for 24 h, and the purity was higher than 95%. G. Choi [25] reported that oxidized components can completely react to form pure ternesite during 10 days in a closed system using stoichiometric amounts of belite and calcium sulfate at 1075 °C. T. Hanein [18] presented an approach to synthesize ternesite with a high purity of 99% by sintering stoichiometric amounts of the produced belite and CaSO_4_ at 1175 °C for 3 days. Liu L. et al. [26] found that the optimum sintering temperature for the formation of high-purity ternesite was about 1200 °C for 12 h by controlling the fineness of the raw material (especially SiO_2_), and the purity could be as high as 96.3% in a closed system.

However, the sintering mechanism and formation kinetics of ternesite have not been systematically studied. In this paper, the formation of ternesite from β-C_2_S and CaSO_4_∙2H_2_O was investigated in detail through isothermal treatment by mixing synthetic β-C_2_S and CaSO_4_∙2H_2_O from 1100 to 1200 °C.

## 2. Materials and Methods

### 2.1. Raw Materials and Samples Preparation

**Figure 1:**
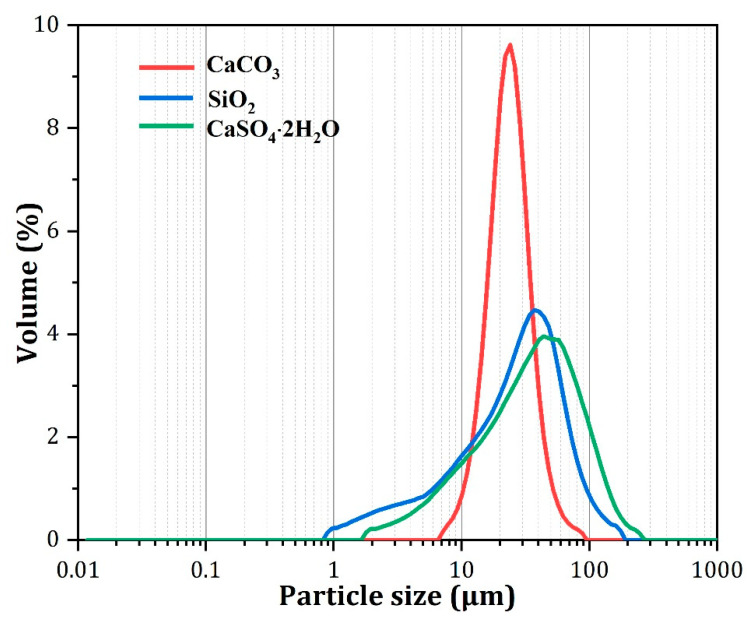
Particle size distribution curves for the raw materials.

**Figure 2:**
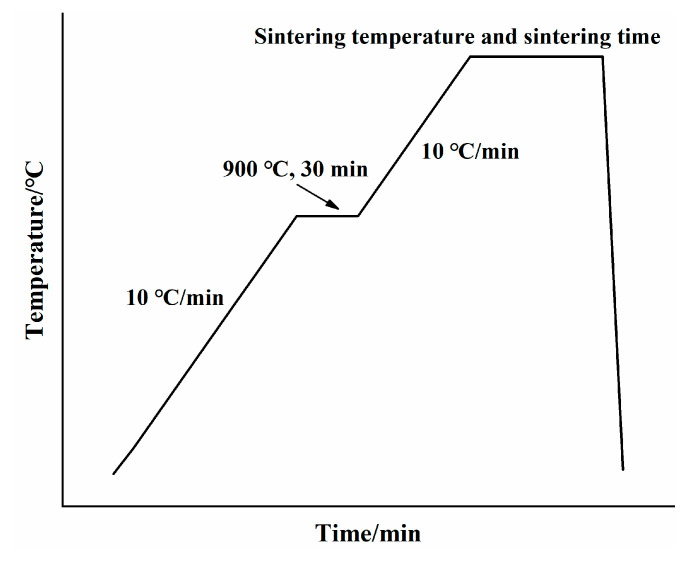
Sintering system of clinker.

**Figure 3:**
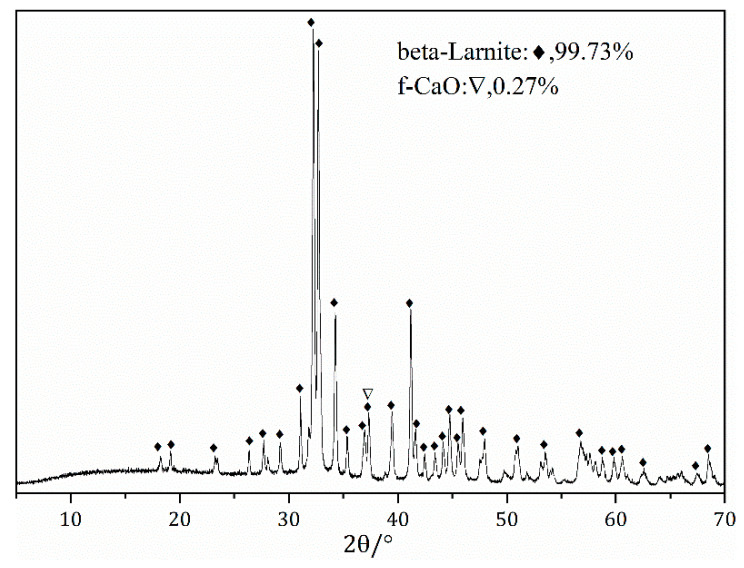
XRD pattern for the synthetic β-C_2_S as a raw material for the preparation of ternesite.

The starting materials used in this study were high-purity calcium carbonate (CaCO_3_), quartz (SiO_2_) and calcium sulfate dihydrate (CaSO_4_∙2H_2_O), all from Sinopharm Chemical Reagent Co., Ltd. (Dongguan, China). Table 1 indicates the chemical composition of the starting materials used for the preparation, showing the high purity of the raw materials used. The particle size distribution (PSD) of the raw powder was obtained using a Microtrac S3500 laser size analyzer (Malvern). Before measurement, the powder (approximately 80 mg) was added to 20 mL of anhydrous ethanol, and the mixture was ultrasonicated for 1 min to disperse the solid particles. Table 2 shows the physical characteristics of these raw materials. Figure 1 shows the results of the particle size distributions (PSDs) for the three raw materials. The chemical reagents were weighed accurately in accordance with the stoichiometric ratio of 2CaO∙SiO_2_. One percent B_2_O_3_ (mass % of the total sample) was added to stabilize the β-C_2_S. The mixture of CaCO_3_, SiO_2_, and B_2_O_3_ was homogenized through ball milling for 12 h. Then, 5% water by weight was added to the mixture. The mixture was pressed into disks, which were oven-dried for 2 h at 105 °C. The dried disks were heated in a resistance furnace from 30 to 1450 °C. During calcination, the pressed sample cake was placed in the sealed Al_2_O_3_ crucible and then put into the electric furnace together with the heating rate of 10 °C/min. In order to promote the full decomposition of calcium carbonate, the temperature is kept for 30 min when the temperature reaches 900 °C. Heating continued to the specified temperature, after which point it was kept warm for a period of time. Take it out after firing and cool it with a fan to get the clinker sample (Figure 2). After sintering at 1450 °C for 2 h, the disks were removed from the resistance furnace and cooled. The purity of the β-C_2_S was determined using the Rietveld method; its f-CaO content was 0.15% (Figure 3). The synthesized C_2_S was ground to a fineness of 200 mesh for the preparation of ternesite.

Then, the sieved C_2_S and CaSO_4_∙2H_2_O were proportioned in a molar ratio of 2:1 and mixed well. The mixture was pressed into disks. The disks were heated to 1100, 1125, 1150, 1175, and 1200 °C. At every temperature, the disks were sintered for 0, 0.25, 0.5, 0.75, 1, 2, 4, 8, 10, or 12 h. Then, the sintered disks were removed from the resistance furnace and cooled immediately.

### 2.2. Characterization

#### 2.2.1. X-ray Fluorescence (XRF) Analysis

The chemical composition of raw materials was analyzed by X-ray fluorescence (XRF) analysis. The samples were tested using a Philips PW2400 XRF spectrometer, and the data were analyzed using UniQuant software. The samples were analyzed for loss on ignition at 1100 °C.

#### 2.2.2. Particle Size Distribution (PSD) Analysis

The particle size distribution (PSD) of the raw powder was obtained using a Microtrac S3500 laser size analyzer (Malvern). Before measurement, the powder (approximately 20 mg) was added to 20 mL of anhydrous ethanol. Each raw material was measured ultrasonically twice, and the average value was taken as the final data.

#### 2.2.3. X-ray Diffraction (XRD) Data Record and Analysis

To determine the phase composition of the samples, the X-ray powder diffraction (XRPD) test was executed using a Rigaku Miniflex 600 with CuKa radiation (λ = 0.15405 nm). The measurements were performed from 5° to 70° (2θ) with a 0.01° step size and 0.2 s dwelling time. The qualitative analysis was performed with the software Search Match. On the basis of phase determination, the phase was quantified by HighScore Plus software with Rietveld refinement based on the following structural model: C_5_S_2_$ [27] (ICSD 85123), β-C_2_S [28] (ICSD 81096), and C$ [29] (ICSD 1956). The refinement parameters included background parameters, phase scaling factors, cell parameters, zero shifts, peak pattern parameters, and orientation corrections. The calculation formula used for quantitative analysis is as follows [30,31]:

(1) $$W_{\alpha }=\frac{S_{\alpha }\rho_{\alpha }V_{\alpha }^{2}\mu_{s}}{G}$$

where *G* is the *G* value of the given standard material; because the alumina standard has the characteristics of amorphous face orientation and very small grains, the standard material is α-Al_2_O_3_, and the measured *G* value is 3.69 × 10^−44^. The *μ_s_* is used to determine the mass attenuation coefficient of the sample. According to the oxide content of the sample given in X-ray fluorescence (XRF), the *μ_s_* of each sample was calculated. *S_α_, V_α_,* *ρ_α_,* and *W_α_* are the scale factor, volume, density, and mass fraction of the measured substance, respectively.

#### 2.2.4. Thermogravimetric–Differential Scanning Calorimetry Analysis

To investigate the physical-chemical reactions during heating of the mixture of β-C_2_S and CaSO_4_∙2H_2_O, thermogravimetric–differential scanning calorimetry (TG-DSC) was carried out on a Mettler Toledo TG/DSC 1600LF comprehensive thermal analyzer. The mixture was heated from 50 to 1300 °C at a speed of 10 °C per minute under an atmosphere of N_2_.

#### 2.2.5. Backscattered Scanning Electron Microscopy (BSE) Analysis

Backscattered electron images (BSE) can be used to analyze the physical phase composition of the specimen. The specimen needs to be treated prior to the test by casting and polishing it, followed by a conductive treatment using the metal coating method. The instrument used for this test is a Japan Electronics JSM-5900 with a tungsten filament, operating voltage of 15 kV, and an electron beam size of 30 nm.

## 3. Results and Discussion

### 3.1. The Physical-Chemical Reaction of the Mixture of β-C_2_S and CaSO_4_∙2H_2_O

**Figure 4:**
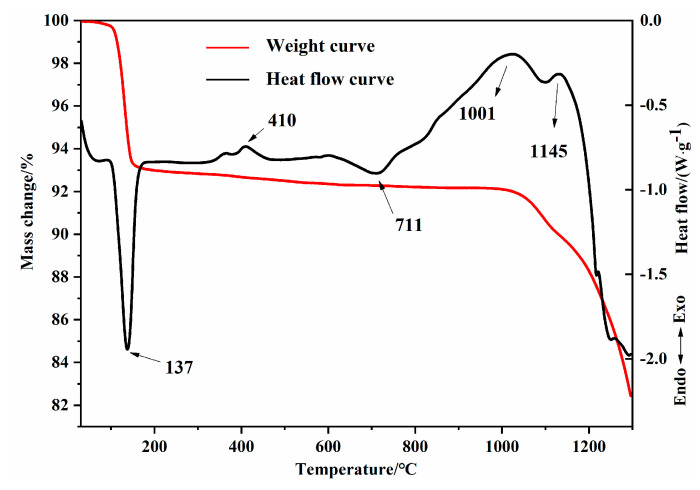
TG-DSC curve of raw materials for C_5_S_2_$ during the heating process.

To investigate the sintering temperature range, we performed thermogravimetric–differential scanning calorimetric (TG-DSC) analysis of the homogeneously mixed raw materials by using a TGA/DSC1600 LF synchronous thermal analyzer. Figure 4 shows the TG-DSC curves of the mixture calcined at temperatures ranging from 50 to 1300 °C. Combining the TG results showed that the endothermic peak at approximately 137 °C corresponded to the dehydration of CaSO_4_∙2H_2_O. It was reported that anhydrous gypsum converted into soluble gypsum, resulting in an exothermic peak at approximately 410 °C [32]. Moreover, the transformation of β-C_2_S into α′-C_2_S caused an endothermic peak at approximately 700 °C [33]. Ternesite could be stable at temperatures ranging between 1000 and 1300 °C [18,23,26,34]; thus, the exothermic peak at approximately 1000 °C indicated the formation of ternesite. The highest ternesite content could be achieved at temperatures between 1100 and 1200 °C [34], and the formation reaction of calcium sulfosilicate was exothermic [19]. Although it was previously reported [35] that calcium sulfate starts decomposing above 1100 °C in an oxidizing atmosphere, the decomposition reaction of calcium sulfate is endothermic [36]. Hence, the exothermic peaks at 1001 and 1145 °C were attributed to the formation of calcium sulfosilicate. As reported by Diouri A. et al. [22], the endothermic peak above 1200 °C and the mass loss may be ascribed to the decomposition of calcium sulfosilicate and CaSO_4_. Consequently, the required calcination temperature range must be between 1000 and 1200 °C for the formation of calcium sulfosilicate. The weight decreases, so we still need to see whether SO_3_ volatilizes. According to the XRF results, the volatilization of SO_3_ can be clearly seen in the oxide composition of raw material and clinker heated to 1300 °C, which verifies our conjecture above.

### 3.2. Factors That Influence of the Formation of Ternesite

**Figure 5:**
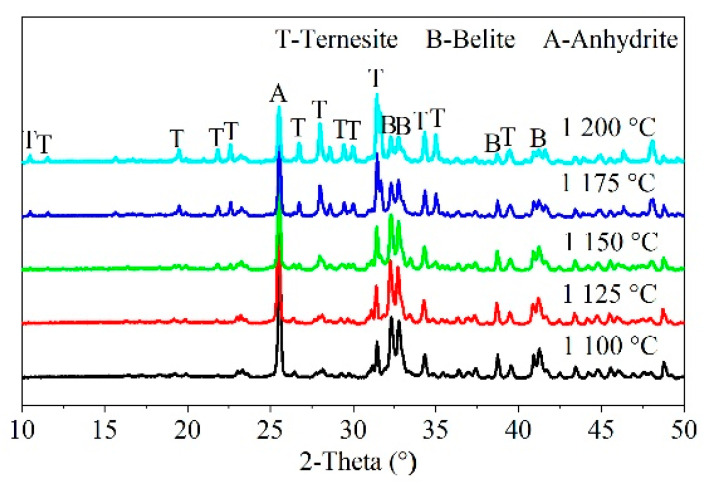
XRD patterns of samples at different temperatures (°C) without holding time.

Temperature is crucial to solid-state reactions. Figure 5 shows the XRD patterns of samples calcined at different temperatures without holding time. The XRD peaks were similar for the samples calcined at 1100, 1125, 1150, 1175, and 1200 °C. Ternesite formed at 1100 °C. The phases in all the samples included ternesite, C_2_S, and CaSO_4_ (C$).

**Figure 6:**
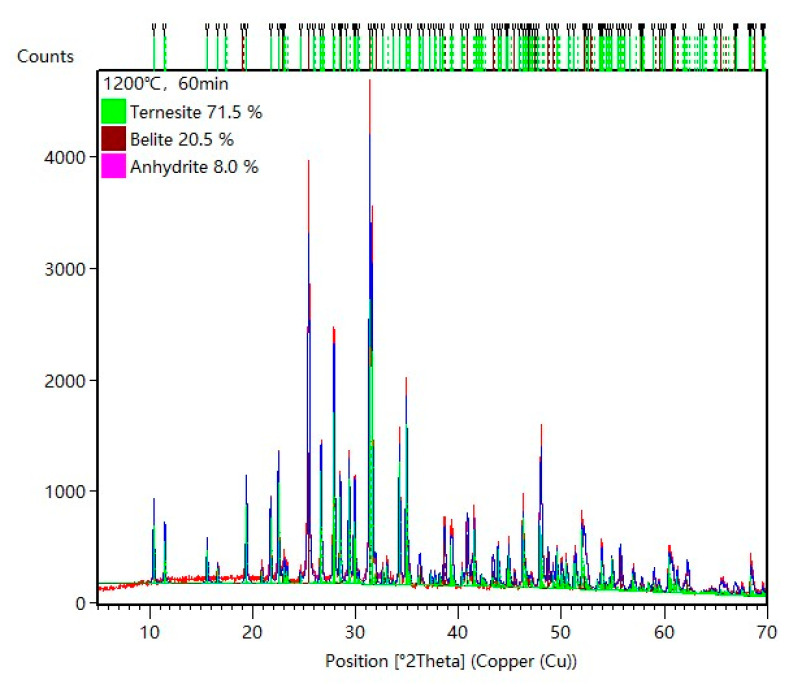
Rietveld refinement of XRD pattern for the sample calcined at 1200 °C for 60 min.

**Figure 7:**
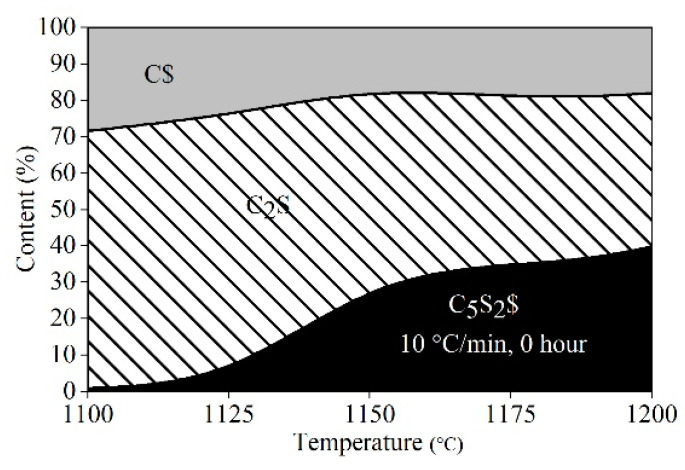
Area plot of the phase composition for the sintering of the raw mixture of beta-C_2_S and CaSO_4_∙2H_2_O at temperatures from 1100 °C to 1200 °C without holding time.

The strength of the diffraction peaks of ternesite increased, and that of the corresponding reactants (C_2_S and CaSO_4_) decreased, with increasing calcination temperature. To determine the quantities of each phase in the calcined samples, the XRD pattern of every sample was fitted and refined by HighScore Plus. Figure 6 presents the fitted plot of the sample calcined at 1200 °C for 1 h to exhibit the accuracy of the refinement. Based on the refinement results, the quantity of each phase was calculated according to Equation (1) and is given in Figure 7. The quantities of each phase varied with the calcination temperature. The quantities of ternesite were greatly enhanced by increasing the calcination temperature. At the same time, the quantities of the reactants decreased as they were consumed. However, the content of the formed ternesite was only approximately 30% at 1200 °C. Ternesite decomposed above 1200–1280 °C to C_2_S and CaSO_4_ [20]. Therefore, it is difficult for ternesite to form completely by only enhancing the temperature. This result illustrates that increasing the calcination temperature can promote the formation of ternesite. However, CaSO_4_ and C_2_S cannot wholly react to form ternesite between 1100 and 1200 °C without preserving heat.

**Figure 8:**
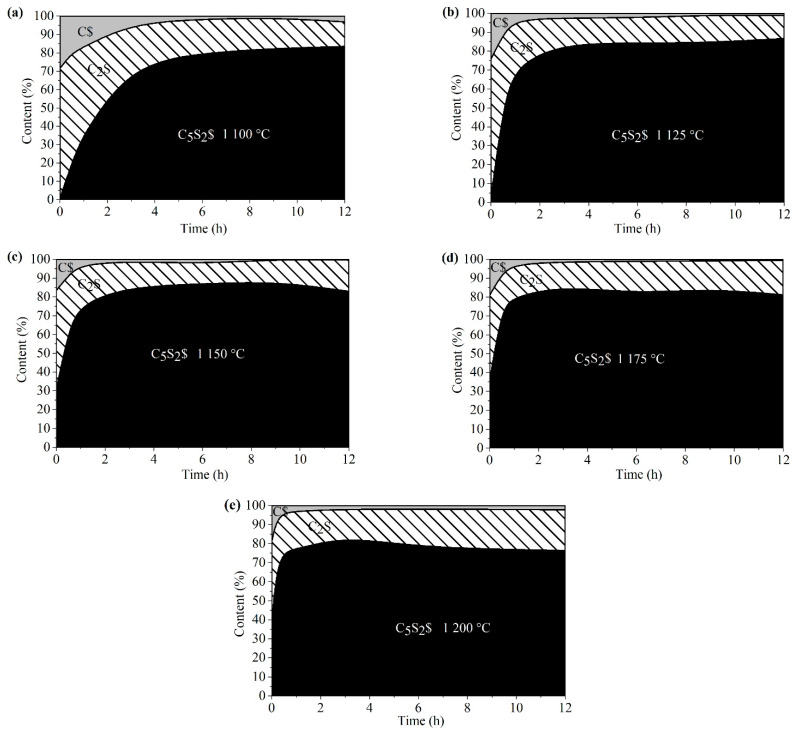
Area plot of the phase composition for the sintering of the raw mixtures of beta-C_2_S and CaSO_4_∙2H_2_O at 1100 °C (**a**), 1125 °C (**b**), 1150 °C (**c**), 1175 °C (**d**), and 1200 °C (**e**) with calcining time.

To promote the formation of ternesite, a mixture of C_2_S and CaSO_4_⋅2H_2_O was kept at each temperature for 1, 2, 4, 8, 10, or 12 h. The contents of each phase are given in Figure 8 for all the samples prepared under isothermal conditions. Prolonging the calcining time promoted the formation of ternesite to a statistically significant extent. However, the formation kinetics of ternesite changed with calcining time. The rate of formation of ternesite was much higher at the initial stage than at the advanced stage at each calcination temperature. This rate is mainly determined by the nature of the solid-state reaction. During 12 h of calcining, CaSO_4_ and C_2_S did not react completely to form ternesite. The maximum quantities of ternesite were almost the same, more than 70% at each calcination temperature. However, the desired calcining time to obtain the maximum quantities of ternesite was different at each calcination temperature. As the calcination temperature increased, the required holding time was shortened. For example, 5 h of holding time was required at 1100 °C for the maximum quantities of ternesite, whereas 1 h was needed at 1200 °C. The higher the calcination temperature is, the quicker the initial formation rate of ternesite. However, very little ternesite was produced after its quantity reached the maximum value at any calcining temperature. The formation of ternesite in large amounts suggested that it hindered the reaction of CaSO_4_ and C_2_S.

### 3.3. Formation Mechanism of Ternesite

**Figure 9:**
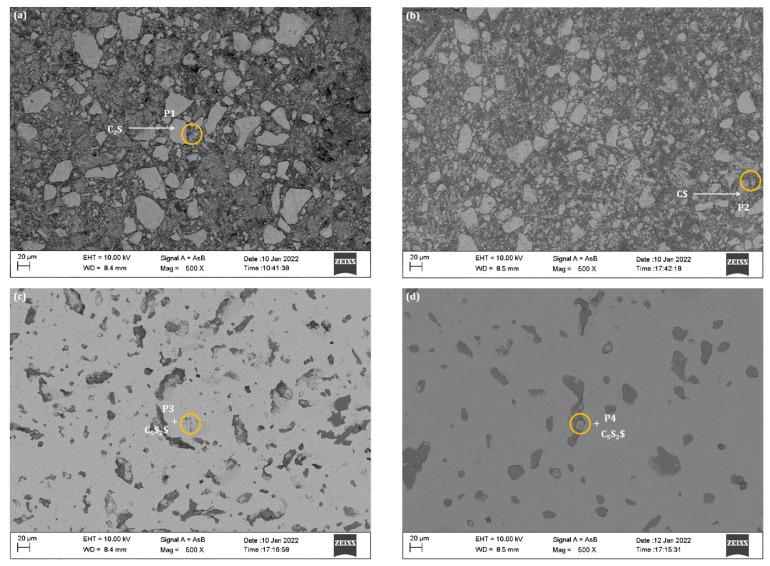
BSE images of the samples: (**a**) is the raw mixture of β-C_2_S and CaSO_4_·2H_2_O, (**b**) is sample treated at 1100 °C for 0 h, (**c**) is sample treated at 1100 °C for 1 h, (**d**) is sample treated at 1100 °C for 12 h. The inset of all EDS analysis represents the spectra of the marked spots in the images, where P1 represents C_2_S, P2 represents CaSO_4_, and P2 and P3 represent C_5_S_2_$. The black area represents the portion of the pore filled with epoxy resin.

**Figure 10:**
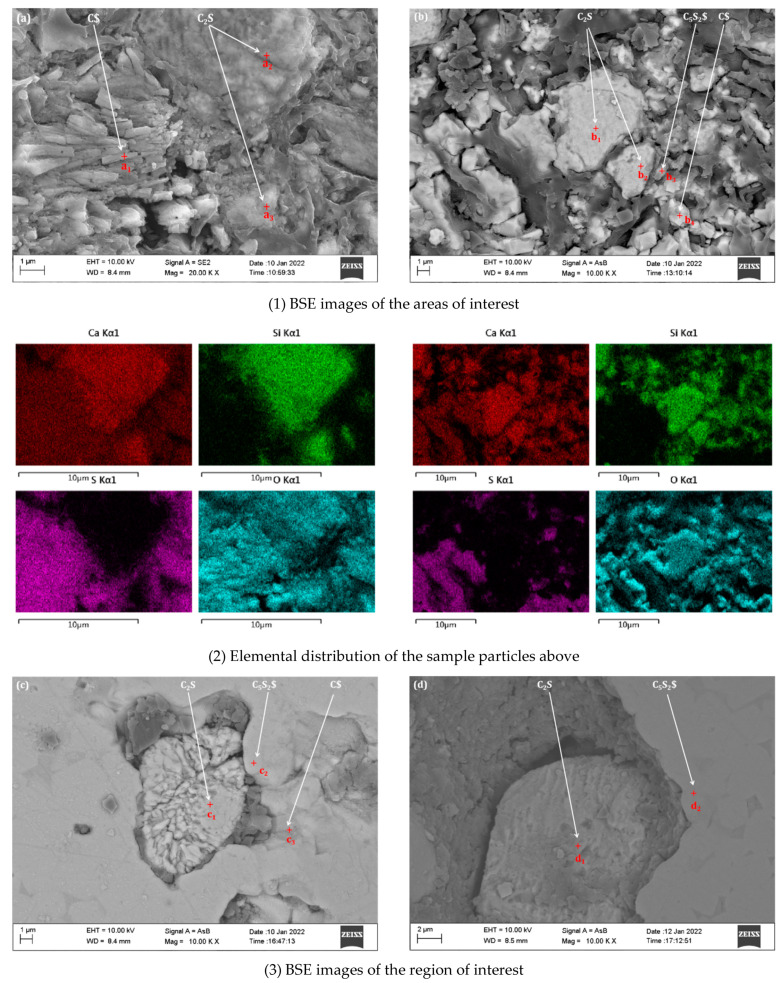

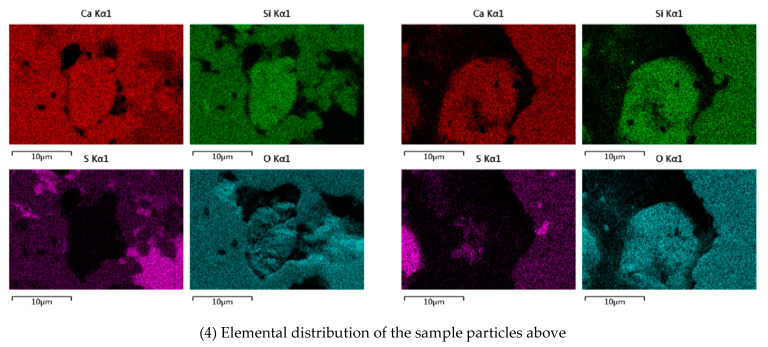
BSE images of the areas of interest: (**a**) is the raw mixture of β-C_2_S and CaSO_4_·2H_2_O, (**b**) is sample treated at 1100 °C for 0 h. All embedding maps for EDS analysis represent the spectra of the marked points in the images. The black area represents the portion of the pore filled with epoxy resin. BSE images of the areas of interest: (**c**) is sample treated at 1100 °C for 1 h, (**d**) is sample treated at 1100 °C for 12 h. All embedding maps for EDS analysis represent the spectra of the marked spots in the images. The black area represents the portion of the pore filled with epoxy resin.

C_5_S_2_$ is formed at the expense of other phases such as C_2_S and CaSO_4_·2H_2_O. The formation mechanism was further studied by BSE-EDS analysis. Figure 9 and Figure 10 present the results regarding the BSE analyses of ternesite samples calcined at different holding times. First, the distribution between C_2_S and CaSO_4_·2H_2_O in Figure 9a is highly uniform, and the change in C_2_S particle size can be observed. As the samples were calcined at 1100 °C without holding time (see Figure 9b), most C_2_S particles started to be enclosed by particles of other phases, which indicates that the solid-phase reactions occurred between the phases. As the duration increased, a distinct C_5_S_2_$ product layer (see Figure 9c) could be observed around the unreacted C_2_S and continued to form with a longer duration until the entire C_2_S group was wholly superseded by C_5_S_2_$ (see Figure 9d). In the end, different sizes of C_5_S_2_$ aggregates were synthesized, and the porosity of single ore samples obtained was relatively high.

Figure 10 shows an enlarged view of the ternesite formation mechanism, containing more details. The EDS energy spectra of the marked points in Figure 8a and the corresponding elemental distributions confirm the particle composition (where a_1_, a_2_, b_1_, b_2_, c_1_, and d_1_ are C_2_S; a_3_, b_4_, and c_3_ are C$; b_3_, c_2_, and d_2_ are C_5_S_2_$). Table 3 also shows the EDS statistics results for all corresponding spots. In Figure 10a, it is apparent that the C_2_S particles were surrounded by columns of C$ particles. At 1100 °C without duration (see Figure 10b), C_5_S_2_$ was formed, and a reaction circle was further formed around C_2_S with a layered structure centered on C_2_S. The C_5_S_2_$ layer was thinner in the middle, and the C$ layer was thicker in the outer layer. As time increased to 1 h (see Figure 10c), more C_5_S_2_$ particles formed and clustered around the product layer. Therefore, our study and that of previous authors have shown that C_2_S acts as an intermediate phase and further reacts with C$ to form C_5_S_2_$ [37]. Similarly, the formation of C_5_S_2_$ in this sintering process was controlled by the diffusion of Ca^2+^ and SO_4_^2−^ through the product layer to the C_2_S structure, which was mainly composed of C_5_S_2_$ (outer layer) and C_2_S (middle layer). When the duration was extended to 12 h (see Figure 10d), the internal C_2_S and C$ were completely converted to C_5_S_2_$. It is worth noting that these transition textures can only be observed on most large C_2_S particles. Compared with smaller C_2_S particles, C_5_S_2_$ particles were easier to form directly. In summary, the formation of C_5_S_2_$ benefits from the formation of the silicate phase, which is a process that restricts transport.

According to XRD analysis and microstructure observation, the content of ternesite rises with sintering temperature, but C_2_S and CaSO_4_ residues remain at 1200 °C. The presence of these phases results from incomplete solid-state reactions between the reactants. By extending the holding time, the formation of C_5_S_2_$ was also controlled by the diffusion of Ca^2+^ and SO_4_^2−^ through the product layer into the C_2_S structure, which is mainly composed of C_5_S_2_$ (outer layer) and C_2_S (middle layer). When the duration was extended to 12 h (see Figure 9d), the inner C_2_S and C$ were completely transformed to C_5_S_2_$. Compared with smaller C_2_S particles, C_5_S_2_$ particles were easier to form directly. In conclusion, the formation of C_5_S_2_$ benefits from the formation of the silicate phase, which is a process that restricts transport.

### 3.4. Formation Kinetics of Ternesite

#### 3.4.1. Experimental Stage

The kinetic analysis of solid reactions has at least three main phases: (1) experimental collection of data, (2) calculation of kinetic properties of the first-stage data, and (3) interpretation of the significance of the parameters evaluated in the second stage. Motivations for performing such analysis include stopping to predict the behavior under test conditions from the collected results and general theoretical considerations of the factors that determine the thermal stability and/or reactivity of the solid [38].

#### 3.4.2. Computational Stage

These mathematical descriptions of the data are usually performed in the form of “kinetic triads” (i.e., the Arrhenius parameters *A* and *Ea* and the reaction model *f*(α) (see Table 4), also known as the conversion function), which are related to the experimental data as follows [38]:

(2) $$\frac{d_{\alpha }}{d_{t}}=A\;exp\left(-\frac{E_{a}}{RT}\right)f\left(\alpha \right)$$

For non-isothermal data, β = d*T*/d*t* is obtained at a fixed temperature condition, and dα/d*t* in the above equation is replaced by β = dα/d*T*. *A* is the pre-exponential factor, *R* is the molar gas constant (8.314 J/(mol∙K)), and *T* is the temperature. Considering that most of the reactions are located in a narrow temperature interval, the activation energy is considered to be independent of temperature, and the dα/d*t* was substituted into Equation (2) to identify the activation energy (*Ea*).

#### 3.4.3. Interpretation

This last stage is undoubtedly the most difficult, because all the accumulated evidence and the accompanying accumulated uncertainties must be evaluated. An attempt must be made to relate the results of the calculations to the actual sequence of the physicochemical processes occurring, i.e., the reaction mechanism. In general, this relationship can be established only with the help of additional information obtained from microscopic, spectroscopic, and structural studies. A prerequisite for establishing such a relationship is the use of appropriate computational methods for processing experimental data. In order to collect data suitable for adequate kinetic analysis, we have previously performed experimental analysis of isothermal and non-isothermal experiments, as well as analysis of BSE, etc. However, experimental data for different warming rates were not collected by us, which may have had an impact on the results of the experiments.

2Ca_2_SiO_4_ + CaSO_4_∙2H_2_O→Ca_5_(SiO_4_)_2_SO_4_ (3)

**Figure 11:**
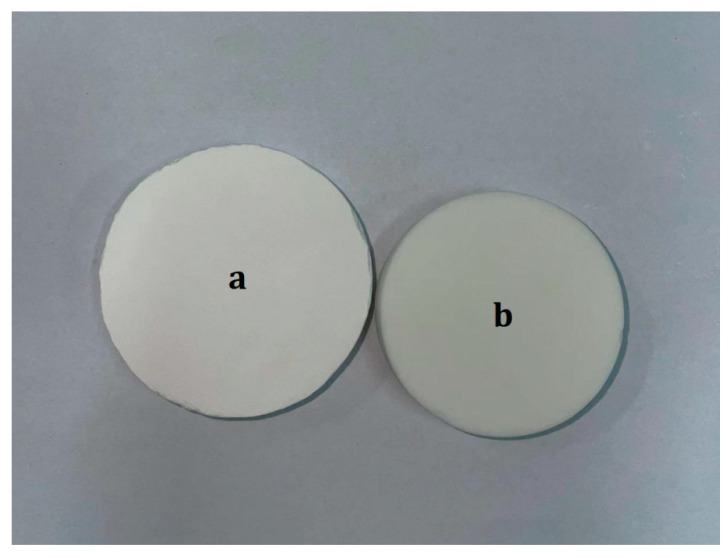
Comparison diagram of raw and clinker.

According to the BSE conclusion, the formation of C_5_S_2_$ was also controlled by the diffusion of Ca^2+^ and SO_4_^2−^ through the product layer into the C_2_S structure, which is mainly composed of C_5_S_2_$ (outer layer) and C_2_S (middle layer). We reviewed the relevant literature [39] and found that the formation of C_4_A_3_$ has a similar structure, as Ca^2 +^ and SO_4_^2−^ also migrate to the product layer, and their model is the Jander diffusion model. For studying the formation kinetics of solid-phase reactions, knowing what has been reported before [38], we need to pay attention to many factors to ensure the accuracy of the experiment. Firstly, for practical experiments, there are two ways to obtain consistent isothermal and non-isothermal data acquisition for the kinetic triplet: (i) isothermal and non-isothermal experiments do not have the same temperature range; (ii) true isothermal conditions cannot be achieved in the very low (<0.02) and very high range (>0.98) of the reaction degree α. The experimental α of this paper does not exceed 0.97. Moreover, for isothermal experiments occurring in the range of non-isothermal experiments, especially in multi-step processes, additional physical phenomena such as melting, polymorphic transitions, sublimation, and evaporation of liquids formed during melting can affect the correct description of the entire temperature range. In addition, a single heating rate is used in this paper, so there are some limitations. The experiment in this paper purely used a diffusion contraction model, as in Figure 11. In Figure 11, a is uniformly mixed raw material, and b is sintered for 12 h. Therefore, this article does not need to consider this factor. Since the experiment is a two-step synthesis, no intermediate phase is generated, which also greatly reduces the difficulty of model building (Equation (3)).

#### 3.4.4. The ICTAC Kinetics Project

(1) Kinetic response model

Most solid-phase reactions are performed by measuring the mass (or mass fraction) of reactants (or products), and reactivity is determined by the following definition:

(4) $$\alpha =\frac{m_{0}-m_{t}}{m_{0}-m_{\infty }}$$

Inside the equation, *m*_0_ is the initial weight percent, *m*_*t*_ is the weight percent at moment *t*, and *m*_∞_ is the ultimate (theoretical) weight percent. The models taking into account the solid-state reactions are presented in Table 4 [40,41].

(2) Selection of reaction models

The selection of kinetic models was usually based on the comparison of the fit of data from different models (Table 4) and the standard deviation of the fit results, combined with modern analytical testing techniques such as microscopic analysis, wave spectroscopy, XRD, product analysis, and analysis of generated gases [42]. These selection criteria were the R^2^ values. Among all the models listed in Table 4, only the diffusion model seems to be consistent and in agreement with the findings of BSE images (see Figure 10). It should be noted that due to the similarity of these models to some extent, it may be difficult to select a suitable reaction model based on data fitting for reactions with a degree of response α < 0.7 [43].

Among these reaction models, the most commonly used is the Jander model (D3), which can be used to describe the reactions between powders, especially some larger powder particles [44,45]. The first-level reaction model (F1) gives a better fit only when the particle size of the reactants is less than 3 µm. It has also been pointed out in the literature that there is a need to maintain consistency in granularity of the reactants when performing solid-phase reaction kinetics experiments to ensure a narrow particle size distribution for the reaction [46], but in practice, this condition is generally difficult to achieve [47]. A wide particle size distribution of reactants can affect the reaction rate [48], but the activation energy is not affected by the change in particle size when isothermal experiments are performed, provided that the experimental conditions are strictly controlled [45].

The expressions of these diffusion models are extremely similar, making them difficult to distinguish [46]. Regarding the correlation coefficients (R^2^) for the D1, D2, D3, and D4 models, we present them in Table 5. Within these models, the Jander model produced the highest R2 values and the smallest standard deviations in the formation reactions (see Table 5). In summary, the Jander model is the most suitable for our kinetic equations.

Obtaining the correct Ea value is possible regardless of the presence of PSD, and in the calculation of the apparent rate constant k, a certain range of reactivity data needs to be chosen to ensure a good linearity of the kinetic model for the reaction time t [45,49,50]. In the present study, the model was fitted with different ranges of reactivity data. The perfect linearity of the fit was determined (most fits were carried out at over 3 points).

**Figure 12:**
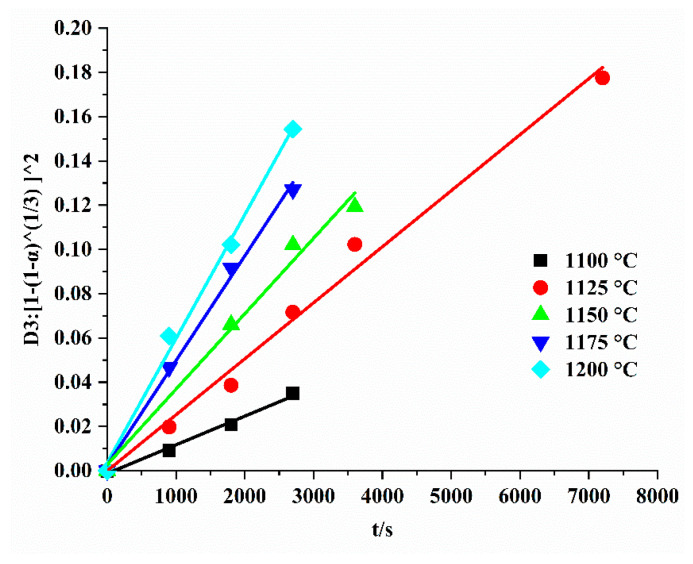
Linear regression lines between [1−(1−α)^(1/3)]^2 and t.

**Figure 13:**
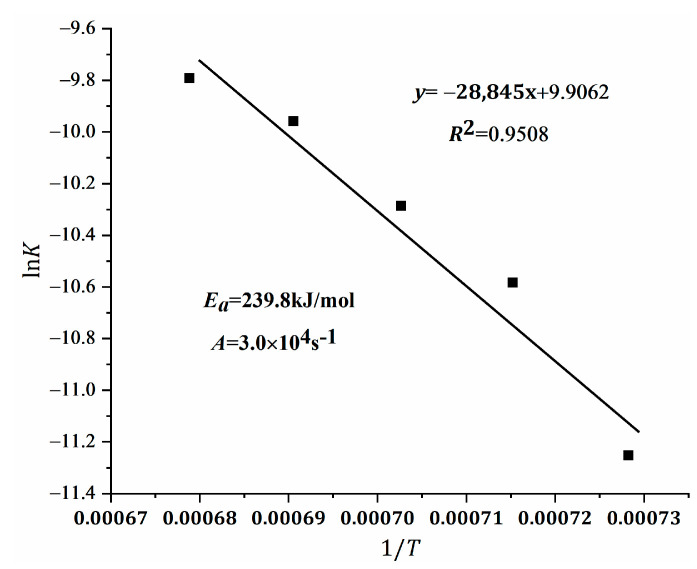
Regression lines between ln*k* and 1/T.

Only temperatures below 1200 °C are compatible with kinetic formation (see Figure 8). In this range, the acquired kinetic accessories are shown in Figure 12 and Figure 13. The illustrations indicate the Arrhenius fit of the reaction constant. Moreover, the activation energy (Ea) was calculated to be 239.8 kJ/mol for the formation of ternesite, and the preexponential factor (A) was 3.0 × 10^4^ s^−1^ (see Figure 11 and Figure 12). The Ea of the formation of C_5_S_2_$ had not been reported yet regardless of the “one-step” or “two-step” method. In their paper, Hauke and colleagues [37] reported that the edge of C_2_S formed by the reaction of SiO_2_ and CaO represents a barrier between the reactants and that the further formation of C_2_S must proceed through this barrier layer of the SiO_2_ structure by Ca^2+^ diffusion. Likewise, the further formation of C_5_S_2_$ during this sintering process is governed by the diffusion of Ca^2+^ and SO_4_^2−^ through the product layers into the SiO_2_ structure, which consists of the predominantly formed C_5_S_2_$ (external) and C_2_S (intermediate). At the same time, diffusion models have been widely applied to the formation kinetics of intermediate silicate phases [51,52,53], suggesting some clues to the kinetics of C_5_S_2_$ formation from C_2_S and C$.

## 4. Conclusions

The formation of C_5_S_2_$ from β-C_2_S and CaSO_4_∙2H_2_O by sintering in the temperature range from 1100 to 1200 °C is described by the following equation:

In the temperature range from 1100 to 1200 °C, both the increase in sintering temperature and the extension of holding time promoted the formation of ternesite. The formation of C_5_S_2_$ is facilitated by the formation of the intermediate silicate phase, which is a process that restricts transport. After 12 h of holding at 1100 °C, the purity of the sample reached 95.3%. However, it is difficult to synthesize pure ternesite in a time period of 12 h. These conclusions can be verified by microstructural observations using EDS mapping of BSE micrographs.

By extending the holding time, the formation of C_5_S_2_$ was also controlled by the diffusion of Ca^2+^ and SO_4_^2−^ through the product layer into the C_2_S structure, which is mainly composed of C_5_S_2_$ (outer layer) and C_2_S (middle layer). The outermost layer contains the C_5_S_2_$ stage, and the composition growth depends on the relationship between the C_2_S and the CaSO_4_ stages; C_5_S_2_$ is formed by the diffusion of Ca^2+^ and SO_4_^2^ from the CaSO_4_ phase to the C_2_S phase. Finally, the formation rate of ternesite could be simply described by the geometrically contracted Jander’s model. This model provides insight into the relationship between beta-dicalcium silicate and calcium sulfate consumption grains during clinking and ternesite formation.

The results reported in this paper provide an in-depth understanding and a phenomenological description of ternesite solid-state formation, which can help cement developers find alternatives to expensive aluminum resources (CSA). The belite–calcium sulfoaluminate–calcium sulfosilicate (BYT) cement system has the advantages of lower CO_2_ emission and a lower firing temperature, that is, low energy consumption. Finally, this study can give some guidance to subsequent researchers in the laboratory for the synthesis of BYT system cement.

Ternesite formation was associated with solid-state reactions, and the formation velocity was controlled by diffusion in the temperature range from 1100 to 1200 °C. The kinetic equation was confirmed by the D3 diffusion model, which was 1 − 2α/3 − (1 − α)^2/3^ = kt. The activation energy of ternesite formation was calculated to be 239.8 kJ/mol. In the future, we will study the formation kinetics of ternesite at different heating rates to further improve the integrity of the experiment.

## Figures and Tables

**Table 1:** Chemical composition (XRF) of raw materials used to prepare ternesite, expressed as wt% of oxide; LOI = loss of ignition at 1100 °C. When the total content (except LOI) is not equal to 100 wt%, the remaining 0.01 wt% is the total content of other trace oxides.

	f-CaO	SiO_2_	Al_2_O_3_	Fe_2_O_3_	SO_3_	Na_2_O	SrO	MgO	TiO_2_	P_2_O_5_	Sum	LOI
CaCO_3_	99.74	0.03	0.05	0.02	0.00	0.04	0.03	0.04	0.02	0.02	99.99	43.10
SiO_2_	0.01	99.80	0.03	0.01	0.00	0.04	0.04	0.03	0.03	0.00	99.99	0.01
CaSO_4_∙2H_2_O	42.93	0.06	0.01	0.00	56.93	0.02	0.03	0.02	0.00	0.00	99.99	20.60

**Table 2:** Physical characteristics of the raw materials.

Raw Material	Calcium Carbonate	Quartz	Calcium Sulfate Dihydrate
Mineralogical analysis ^(a)^	CaCO_3_	SiO_2_	CaSO_4_·2H_2_O
Particle size ^(b)^ d_10_ (μm)	13.76	4.49	7.81
d_50_ (μm)	22.46	26.59	34.65
d_90_ (μm)	36.38	64.32	92.71

^(a)^ XRD phase identification. ^(b)^ Laser diffraction granulometry.

**Table 3:** EDS spectral results of the marked spots in the image.

	Spots	P1	P2	P3	a_1_	a_2_	a_3_	b_1_	b_2_	b_3_	b_4_	c_1_	c_2_	c_3_	d_1_	d_2_
At%																
Ca/Si		2.0		2.4	2.0	2.0		2.1	1.9	2.5		2.0	2.4		2.2	2.4
Ca/S			1.0	5.0			1.0			5.2	1.0		5.0	1.0		5.0
Si/S				2.2						2.2			2.1			2.1

**Table 4:** Solid-state rate and integral expressions for different reaction models [39,40,41].

Model	Differential Form f(α) = kt ^(a)^	Integral Form g(α) = kt	Model Type
Power law (P2)	2α^1/2^	A^1/2^	Nucleation and growth models
Power law (P3)	3α^2/3^	A^1/3^	
Power law (P4)	4α^3/4^	α^1/4^	
Avrami–Erofeyev (A2)	2(1 − α)[−ln(1 − α)]^1/2^	[−ln(1 − α)]^1/2^	
Avrami–Erofeyev (A3)	3(1 − α)[−ln(1 − α)]^2/3^	[−ln(1 − α)]^1/3^	
Avrami–Erofeyev (A4)	4(1 − α)[−ln(1 − α)]^3/4^	[−ln(1 − α)]^1/4^	
Contracting area (R2)	2(1 − α)^1/2^	1 − (1 − α)^1/2^	Geometrical contraction models/Phase boundary control
Contracting area (R3)	3(1 − α)^2/3^	1 − (1 − α)^1/3^	
1-D diffusion (D1)	1/(2α)	α^2^	Diffusion models
2-D diffusion (D2)	−[1/ln(1 − α)]	(1 − α)ln(1 − α) + α	
3-D diffusion (D3)	[3/(1 − α)^2/3^]/[2(1 − (1 − α)^1/3^)]	[1 − (1 − α)^1/3^]^2^	
3-D diffusion (D4)	3/[2(1 − α)^−1/3^ − 1]	1 − 2α/3 − (1 − α)^2/3^	
Zero-order (F0/R1)	1	α	Reaction-order models
First-order (F1)	1 − α	−ln(1 − α)	
Second-order (F2)	(1 − α)^2^	[1/(1 − α)] − 1	
Third-order (F3)	(1 − α)^3^	(1/2) [(1 − α)^−2^ − 1]	

^(a)^ k is the slope of the kinetic equation, t is the reaction time, and α is the degree of reactivity.

**Table 5:** Comparison of correlation coefficient (*R*^2^) fitted by different kinetic functions.

Temperature/°C	D1	D2	D3	D4
1100	0.9995	0.9420	0.9909	0.9321
1125	0.9333	0.9679	0.9898	0.9779
1150	0.9618	0.9745	0.9822	0.9780
1175	0.8944	0.9266	0.9539	0.9373
1200	0.8716	0.9178	0.9601	0.9342
Average	0.9191	0.9507	0.9749	0.9608
Standard Deviation	0.0458	0.0223	0.0154	0.0213

## Data Availability

Not applicable.

## References

[1] Monteiro P.J.M., Miller S.A., Horvath A. (2017). Toward sustainable concrete. Nat. Mater..

[2] Gartner E. (2004). Industrially interesting approaches to “low-CO_2_” cements. Cem. Concr. Res..

[3] Gartner E.M., Li G.S. (2006). High Belite Sulfoaluminate Clinker: Fabrication Process and Binder Preparation. World Patent.

[4] Hanein T., Galan I., Elhoweris A., Khare S., Skalamprinos S., Jen G., Whittaker M., Imbabi M.S., Glasser F.P., Bannerman M.N. (2016). Production of belite calcium sulfoaluminate cement using sulfur as a fuel and as a source of clinker sulfur trioxide: Pilot kiln trial. Adv. Cem. Res..

[5] Lv L., Šavija B., Li L., Cui H., Han N., Xing F. (2021). Prehydration of calcium sulfoaluminate (CSA) clinker at different relative humidities. Cem. Concr. Res..

[6] Beretka J., Vito B.D., Santoro L., Sherman N., Valenti G.L. (1993). Hydraulic behaviour of calcium sulfoaluminate-based cements derived from industrial process wastes. Cem. Concr. Res..

[7] Beretka J., Vito B.D., Santoro L., Sherman N., Valenti G.L. (1993). Utilisation of industrial wastes and by-products for the synthesis of special cements. Resour. Conserv. Recycl..

[8] Belz G., Beretka J., Marrocoli M., Santoro L., Sherman N., Valenti G.L. (1995). Use of fly ash, blast furnace slag and chemical gypsum for the synthesis of calcium sulphoaluminate-based cements. Spec. Publ..

[9] Beretka J., Marrocoli M., Sherman N., Valenti G.L. (1996). The influence of C4A3$ content and w/s ratio on the performance of calcium sulfoaluminate-based cements. Cem. Concr. Res..

[10] Osokin A.P., Krivoborodov Y.R., Dyukova N.F. (1992). Sulfoferrite cements. National Council for Cement and Building Materials.

[11] Adolfsson D., Menad N., Viggh E., Bjorkman B. (2007). Steelmaking slags as raw material for sulphoaluminate belite cement. Adv. Cem. Res..

[12] Pryce M. (1972). Calcium sulphosilicate in lime-kiln wall coating. Min. Mag..

[13] Nievoll J., Jorg K.S., Dosinger J. (2007). Corpus, Sulphur, spurrite and rings-always a headache for the cement kiln operator?. J. Refra Innov..

[14] Brotherton P.D., Epstein J.M., Pryce M.W., White A.H. (1974). Crystal structure of “calcium sulphosilicate”, Ca_5_(SiO_4_)_2_SO_4_. Aust. J. Chem..

[15] Shen Y., Qian J., Huang Y., Yang D. (2015). Synthesis of belite-sulfoaluminate-ternesite cements with phosphogypsum. Cem. Concr. Compos..

[16] Shen Y., Chen X., Zhang W., Li X., Qian J. (2018). Influence of ternesite on the properties of calcium sulfoaluminate cements blended with fly ash. Constr. Build. Mater..

[17] Skalamprinos S., Jen G., Galan I., Whittaker M., Elhoweris A., Glasser F. (2018). The synthesis and hydration of ternesite. Ca_5_(SiO_4_)_2_SO_4_. Cem. Concr. Res..

[18] Hanein T., Galan I., Glasser F.P., Skalamprinos S., Elhoweris A., Imbabi M.S., Bannerman M.N. (2017). Stability of ternesite and the production at scale of ternesite-based clinkers. Cem. Concr. Res..

[19] Skalamprinos S., Galan I., Hanein T., Glasser F. (2018). Enthalpy of formation of ye’elimite and ternesite. J. Therm. Anal. Calorim..

[20] Ukrainczyk N., Frankoviæ Mihelj N., Šipušić J. (2013). Calcium sulfoaluminate eco-cement from industrial waste. Chem. Biochem. Eng. Q..

[21] Jing G., Zhang J., Lu X., Xu J., Gao Y., Wang S., Cheng X., Ye Z. (2020). Comprehensive evaluation of formation kinetics in preparation of ternesite from different polymorphs of Ca_2_SiO_4_. J. Solid State Chem..

[22] Addi K.B., Diouri A., Khachani N., Boukhari A. (2018). Mineralogical stabilization of Ternesite in Belite 285 Sulfo-Aluminate Clinker elaborated from limestone, shale and phosphogypsum. Matec. Web Conf..

[23] Gutt W., Smith M.A. (1966). A new calcium silicosulphate. Nature.

[24] Pliego-Cuervo Y.B., Glasser F.P. (1979). The role of sulphates in cement clinkering: Subsolidus phase relations in the system CaO-Al_2_O_3_-SiO_2_-SO_3_. Cem. Concr. Res..

[25] Choi G., Glasser F.P. (1988). The sulphur cycle in cement kilns: Vapour pressures and solidphase stability of the sulphate phases. Cem. Concr. Res..

[26] Liu L., Zhang W., Ren X., Ye J., Zhang J., Qian J. (2021). Formation, structure, and thermal stability evolution of ternesite based on a single-stage sintering process. Cem. Concr. Res..

[27] Irran E., Tillmanns E., Hentschel G. (1997). Ternesite, Ca_5_(SiO_4_)_2_SO_4_, a new mineral from the Ettringer Bellerberg/Eifel, Germany. Miner. Petrol..

[28] Mumme W.G., Hill R.J., Bushnell-Wye G., Segnit E.R. (1995). Rietveld crystal structure refinements, crystal chemistry and calculated powder diffraction data for the polymorphs of dicalcium silicate and related phases. Neues Jahrb. Mineral. Abh..

[29] le Page Y., Donnay G. (1976). Refinement of the crystal structure of low-quartz. Acta Crystallogr. Sect. B Struct. Crystallogr. Cryst. Chem..

[30] Hill R.J., Howard C.J. (1987). Quantitative phase analysis from neutron powder diffraction data using the Rietveld method. J. Appl. Crystallogr..

[31] De la Torre A.G., Aranda M.A.G. (2003). Accuracy in Rietveld quantitative phase analysis of Portland cements. J. Appl. Crystallogr..

[32] Javangula H., Lineberry Q. (2014). Comparative studies on fire-rated and standard gypsum wallboard. J. Therm. Anal. Calorim..

[33] Yang N., Yue W. (2000). The Handbook of Inorganic Non-Metallic Materials Atlas.

[34] Scholten T. (2017). Reaktionskinetik von Sulfatischen Klinkerphasen in Zementen Mit Verminderter CO_2_-Last. Ph.D. Thesis.

[35] Salman O.A., Khraishi N. (1988). Thermal decomposition of limestone and gypsum by solar energy. Sol. Energy.

[36] Swift W.M., Panek A.F., Smith G.W., Vogel G.J., Jonke A.A. (1976). Decomposition of calcium sulfate: A review of the literature. OSTI.

[37] Hauke K., Kehren J., Bohme N., Zimmer S., Geisler T. (2019). In situ hyperspectral raman imaging: A new method to investigate sintering processes of ceramic material at high temperature. Appl. Sci..

[38] Brown M.E., Maciejewski M., Vyazovkin S., Nomen R., Sempere J., Burnham A.A., Opfermann J., Strey R., Anderson H.L., Kemmler A. (2000). Computational aspects of kinetic analysis: Part A: The ICTAC kinetics project-data, methods and results. Thermochim. Acta.

[39] El Khessaimi Y., El Hafiane Y., Smith A. (2019). Examination of ye’elimite formation mechanisms. J. Eur. Ceram. Soc..

[40] Maciejewski M. (2000). Computational aspects of kinetic analysis: Part B: The ICTAC Kinetics Project—The decomposition kinetics of calcium carbonate revisited, or some tips on survival in the kinetic minefield. Thermochim. Acta.

[41] Khawam A., Flanagan D.R. (2006). Solid-state kinetic models: Basics and mathematical fundamentals. J. Phys. Chem. B.

[42] Khawam A., Flanagan D.R. (2006). Basics and applications of solid-state kinetics: Apharmaceutical perspective. J. Pharm. Sci. Sci..

[43] Sharp J.H., Brindley G.W., Achar B.N.N. (1966). Numerical data for some commonly used solid state reaction equations. Ceram. Soc..

[44] Beretka J. (1984). Kinetic analysis of solid-state reactions between powdered reactants. J. Am. Ceram. Soc..

[45] Li X., Zhang Y., Shen X., Pan Z. (2014). Kinetics of calcium sulfoaluminate formation from tricalcium aluminate, calcium sulfate and calcium oxide. Cem. Concr. Res..

[46] Tagawa H., Igarashi K. (1986). Reaction of strontium carbonate with anatase and rutile. J. Am. Ceram. Soc..

[47] Koga N., Criado J.M. (1998). Kinetic analyses of solid-state reactions with a particle-size distribution. J. Am. Ceram. Soc..

[48] Fatu D. (1991). Factors on which the rate of solid–solid reactions depends. Thermochim. Acta.

[49] Maciejewski M. (1992). Somewhere between fiction and reality. J. Therm. Anal..

[50] Koga N. (1994). A review of the mutual dependence of Arrhenius parameters evaluated by the thermoanalytical study of solid-state reactions-the kinetic compensation effect. Thermochim. Acta.

[51] Bohme N., Hauke M., Neuroth M., Geisler T. (2020). In situ hyperspectral Raman imaging of ternesite formation and decomposition at high temperatures. Minerals.

[52] Mitsuda T., Asami J., Matsubara Y., Toraya H. (1985). Hydrothermal formation of γ-dical siliconicate from lim-silica mix. Cem. Concr. Concr. Res..

[53] Singh N.B. (2006). Hydrothermal synthesis of β-dicalcium silicate (β-Ca_2_SiO_4_). Prog. Cryst. Growth Charact. Mater..

